# Surgical treatment and prognosis of recurrent and radiotherapy insensitive nasopharyngeal carcinoma

**DOI:** 10.1016/j.bjorl.2023.101366

**Published:** 2023-11-20

**Authors:** Yanming Zhao, Jugao Fang, Qi Zhong, Jiamin Zhang, Jiaming Chen, Lizhen Hou, Ru Wang

**Affiliations:** Capital Medical University, Beijing Tongren Hospital, Department of Otorhinolaryngology Head and Neck Surgery, Beijing, China

**Keywords:** Nasopharyngeal carcinoma, Recurrence, Surgery, Nasendoscopy, Prognosis

## Abstract

**Objective:**

To explore the effect of surgical treatment and related prognostic factors for recurrent Nasopharyngeal Carcinoma (NPC) after radiotherapy and the pathological types of nasopharyngeal carcinoma insensitive to radiotherapy.

**Methods:**

A total of 70 NPC patients who underwent surgery at the Department of Otolaryngology, head and neck surgery, from January 2005 to December 2020 were retrospectively included: 41 males and 29 females, aged 21–75 years, 47 patients were pathologically classified as NPC (nonkeratinizing, undifferentiated type), 10 patients as adenoid cystic carcinoma, 13 patients as other types, 45 patients had received radiotherapy preoperatively, and 25 patients had not received radiotherapy preoperatively. All patients underwent surgical treatment under general anesthesia. Fifty-six patients underwent nasoendoscopic NPC resection, seven patients underwent open surgery, and seven patients underwent combined nasoendoscopic and open surgery. The median follow-up was 39 months. Tumor volume, extent of involvement, lymph node metastasis, imaging characteristics, surgical approach and efficacy, postoperative complications, and 2-, 3-, and 5-year postoperative survival rates were calculated for all patients. Statistical analysis was performed using spss22 Kaplan Meier survival analysis and Cox regression analysis were performed.

**Results:**

Among the 70 patients, the overall 2-year survival rate was 93.4%, the 3-year survival rate was 90.8%, and the 5-year survival rate was 80.3%. Multivariate analysis showed that TNM stage and age at onset were independent prognostic factors for NPC outcome.

**Conclusion:**

Depending on the size and location of the tumor, endoscopic surgery, open surgery, and combined open surgery with nasoendoscopy may be considered for recurrent and radiotherapy insensitive NPC.

**Level of Evidence:**

Level 4.

## Highlights

• The treatment of recurrent or some pathological types of NPC is full of challenges.
• The surgical treatment has a good therapeutic effect on NPC.

## Introduction

Nasopharyngeal Carcinoma (NPC) is a head and neck malignant tumor located in the nasopharynx, and its primary site is mostly found in the apical posterior wall of the nasopharynx and can invade surrounding tissues. According to the World Health Organization (WHO) classification, the pathological types of NPC can be classified as keratinizing squamous cell carcinoma, nonkeratinizing squamous cell carcinoma and basaloid squamous cell carcinoma, adenocarcinoma, adenoid cystic carcinoma, mucoepidermoid carcinoma, as well as malignant pleomorphic adenoma.^1^ There is significant ethnic and regional variation in the incidence of NPC, with more than 70% of newly diagnosed cases reported worldwide in 2018 patients originating from East and South East Asia.^2^ The updated GLOBOCAN database of the World Health Organization 2020 (GLOBOCAN 2020) shows that the global crude incidence of nasopharyngeal carcinoma is 1.7/100,000 in 2020, ranked 22nd in the world incidence of malignancies, and 21st in mortality, as calculated by the model.^3^

Radiotherapy is the accepted treatment of choice for nasopharyngeal carcinoma. In recent years, with the advent and development of Intensity-Modulated Radiation Therapy (IMRT) technology, comprehensive treatment based on IMRT has allowed the 5-year overall survival rate of NPC patients to reach more than 80%, and the local control rate can reach more than 90%. However, 10%–15% of patients still experience tumor recurrence after radiotherapy requiring further treatment.^4^, ^5^ Treatment of recurrent nasopharyngeal carcinoma after radiotherapy most often follows a multidisciplinary comprehensive treatment paradigm, which can be tailored to the individual patient's circumstances and may include: surgical treatment, re-irradiation, chemotherapy, targeted therapy, and a combination of the modalities described above.

In addition, for some rare types of nasopharyngeal pathology such as adenoid cystic carcinoma, mixed neoplastic malignant transformation, mucoepidermoid carcinoma, papillary adenocarcinoma, etc., because of the insensitivity to radiotherapy, comprehensive treatment with surgical treatment is required. Recently, with improved equipment of endoscopic surgery, deeper research on the anatomy related to the skull base of the nasopharynx, and improved surgical techniques, surgical treatment represented by endoscopic sinus surgery has been more used in the treatment of recurrent nasopharyngeal carcinoma and radiotherapy insensitive types of nasopharyngeal carcinoma.^6^, ^7^, ^8^

In this study, 70 patients with recurrent nasopharyngeal carcinoma and radiotherapy insensitive type rhinitis who underwent surgical treatment were reviewed, and their clinical features, surgical treatment, and outcomes were analyzed.

## Methods

### Study population

This retrospective study included seventy patients (41 male and 29 female), aged 48 ± 11 (range, 21–74) years, diagnosed with NPC at the Department of Otolaryngology-Head and Neck Surgery, between January 2005 and December 2020. Surgical resection was recommended by comprehensive multidisciplinary discussion in all cases. The characteristics of our study population are shown in Table 1. The protocol was performed in accordance with the declaration of Helsinki and its later amendments. Informed consent was waived due to an anonymous data extraction with no direct patient and public involvement in the study in our registered databases.

**Table 1:** Patient characteristics.

Cases	Age (years)	Sex	Smoking history	Drinking status	Histotype	T, N, M stage	Adjuvant therapy	Complications	Follow-up (months)
1	56	Male	Yes	Yes	rNPC	T4N2M0	Chemotherapy	Facial sweating, pain when lifting the right hand	48
2	49	Female	No	No	MEC	T2N0M0	None	None	41
3	48	Female	No	No	PA	T3N0M0	None	None	33
4	55	Male	Yes	No	rNPC	T2N0M0	None	None	31
5	37	Female	No	No	rNPC	T2N2M0	None	Diplopia	28
6	63	Female	No	No	MEC	T4N0M0	IMRT	Xerostomia	20
7	53	Female	No	No	rNPC	T4N2M0	None	Nasal blockage, choking on eating	13

rNPC, Recurrent Nasopharyngeal Carcinoma; MEC, Mucoepidermoid Carcinoma; PA, Papillary Adenocarcinoma; IMRT, Intensity-Modulated Radiotherapy.

### Surgical approach

**Figure 1:**
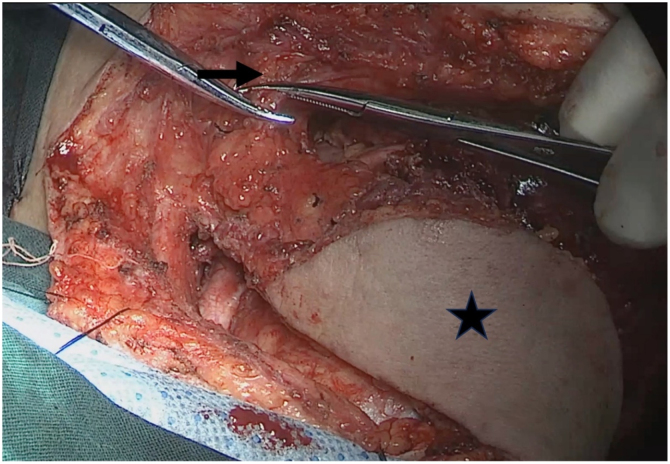
The submental flap was completed, and the distal facial vein of the facial artery was ready to be ligated. Black Arrow: facial artery and vein; Black pentagram: the finished submental flap.

**Figure 2:**
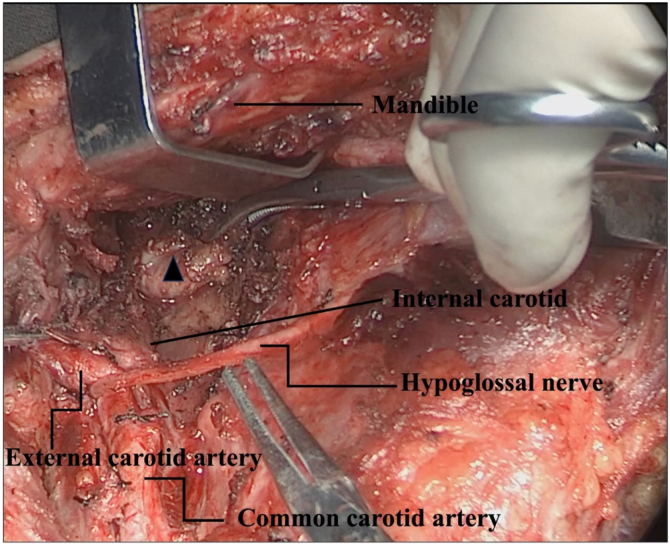
Anatomic exposure of internal carotid artery and resection of metastatic lymph nodes in retropharyngeal space. Black triangle: suspicious metastatic retropharyngeal lymph node.

**Figure 3:**
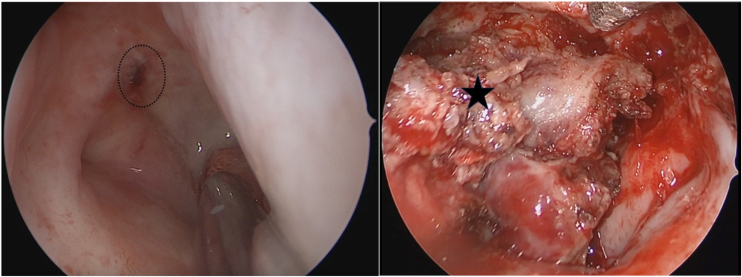
Nasal endoscopic observation of recurrent tumors and tumor resection in nasopharynx. Circle: tumor in the pharyngeal recess; Black pentagram: the boundary of tumor resection.

**Figure 4:**
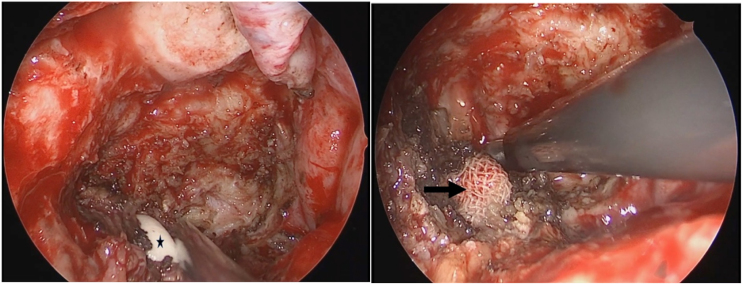
Finger exploration through neck parapharyngeal space to nasopharynx and a a gauze was placed on the inner side of the internal carotid artery to prevent damage to the internal carotid artery during endoscopic removal of tumors. Black pentagram: the surgeon's finger; Black Arrow: the gauze placed through the neck.

**Figure 5:**
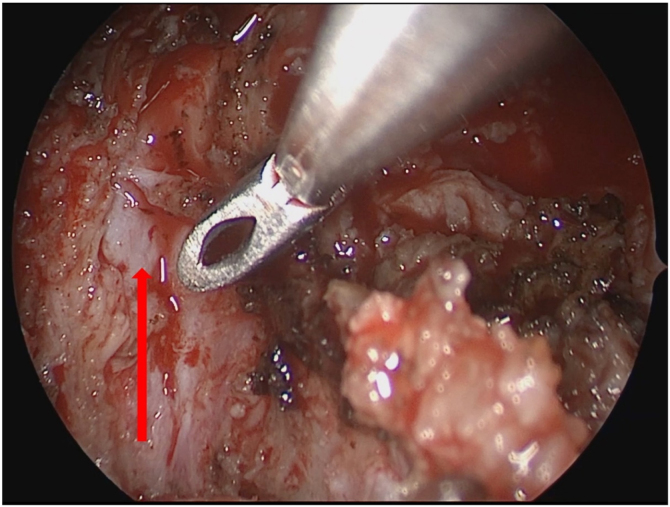
Nasal endoscopy revealed the internal carotid artery exposed at the lateral border of tumor resection. The red arrow: the internal carotid arteryindicates of the parapharyngeal.

**Figure 6:**
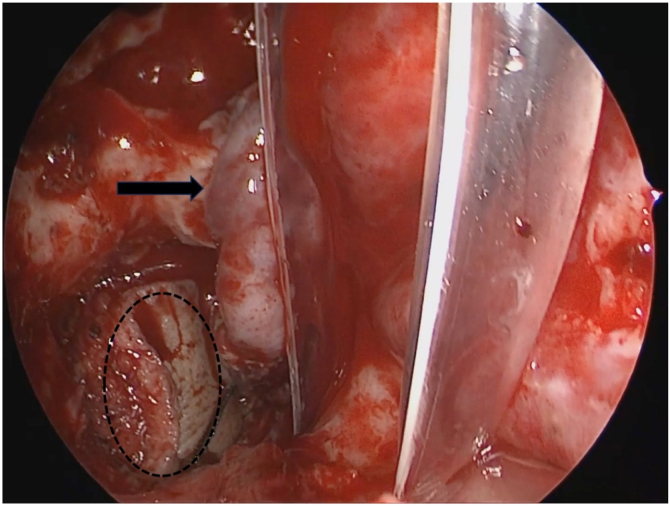
Place the submental flap in the nasopharynx through the neck to protect the internal carotid artery and contralateral nasal septum mucosal flap to repaire exposed sphenoid sinus and skull base bone. Circle, submental flap; Black Arrow, nasal septum mucosal flap.

**Figure 7:**
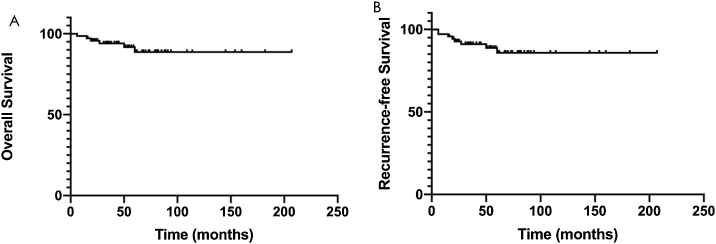
The survival rate (A) and recurrence free survival rate (B) of 70 patients.

All patients underwent preoperative physical examination, nasopharynx MRI, neck CT, nasopharynx CT, and neck ultrasound to evaluate the tumor size, cervical lymph node metastasis, and general condition, and different surgical options were decided by Multidisciplinary Comprehensive Discussion (MDT). Endoscopic NPC resection alone: in all 56 patients, the rt1‒rt3 cases, nasopharyngeal MRI, nasopharynx CT, and neck ultrasonography showed that the lesions were located in the nasopharynx, no cervical lymph node metastasis was observed, and no distant metastasis was observed as assessed by chest CT, abdominal ultrasonography, and PET/CT. The tumor and surrounding 0.5 cm safety margins were completely resected under nasoendoscopy, and intraoperative margins were examined pathologically by frozen section; Nasoendoscopic NPC resection combined with cervical lymph node dissection: cervical lymph node dissection was performed first according to the extent of the lesion, followed by nasoendoscopic NPC resection; Transpharyngeal space nasopharyngeal carcinoma resection: the preoperative TNM stage was T4N1M0 in 1 patient who underwent a transpharyngeal space nasopharyngeal carcinoma resection. Preoperative examination showed enlarged lymph nodes in the parapharyngeal space, intraoperative resection of the skull base lymph nodes in the left parapharyngeal space, left cervical lymph node dissection (Levels I, II, III and VA) with partial resection of the left parotid gland to expose the medial border of the tumor, and oral resection of the tumor using Davis opener; Resection of external circumflex nasopharyngeal carcinoma of the maxilla: nasopharynx was repaired by abdominal full-thickness skin flap in one patient, temporalis muscle flap in one patient, and left forearm free flap in one patient; excision of NPC with mandibular swing approach: 3 cases, the surgical procedure has been reported elsewhere. Combined endoscopic nasopharyngeal resection via the parapharyngeal space approach: submental flap making was performed in six of seven patients, the ipsilateral upper cervical and parapharyngeal space lymph nodes were dissected, the vascular sheath of the parapharyngeal space was dissected superiorly via the cervical parotid approach to the skull base, and the tumor was dissected medially superiorly from the parapharyngeal space to the nasopharynx using the nasoendoscopy, in which the combined internal and external approaches were taken to remove the tumor en bloc. The Figs. 1‒7 illustrate the specific surgical procedure.

### Follow-up

The first postoperative review of the patients was taken as the start of follow-up, and the follow-up period was until April 1, 2021. The condition of the surgical area was checked by first review in the 1ST‒2ND week after operation. For patients requiring radiotherapy, radiotherapy was started around 1 month after surgery. Outpatient review again 1 month after radiotherapy. Thereafter, outpatient reviews were conducted every three months in the 1ST‒2ND postoperative year, every six months in the 2nd‒3rd postoperative year, and annually in the 4TH‒5TH postoperative year. At the time of review, the nasopharynx was examined by fiberoptic nasopharyngoscopy, local conditions by MRI and CT of the nasopharynx, and, if necessary, distant metastases by lung CT or whole-body PET/CT. Among them, patients who could not undergo outpatient review for personal reasons, it was recommended to review them regularly to the local hospital and follow-up the patients by telephone. The median follow-up was 39 months.

### Statistical methods

IBM SPSS 22.0 software was applied for statistical analysis. Statistical methods statistical analysis was performed by Chi-Square test from age (in groups of >60 years), gender, T stage, N stage, surgical approach, type of cases, resection margins, preoperative chemoradiotherapy, postoperative chemoradiotherapy, smoking history, alcohol drinking history. Survival was calculated by plotting survival curves with the Kaplan-Meier method. Multivariate analysis was performed using the Cox regression analysis model; *p* < 0.05 was defined as statistically significant.

### Adjuvant treatment

We followed the Multidisciplinary Comprehensive Therapy (MDT) pattern and chose a rational use of radiotherapy, chemotherapy, immunotherapy, or other means for recurrent and radiotherapy-insensitive NPC postoperatively. We aimed to develop an individualized comprehensive treatment strategy when possible, to improve efficacy while ensuring the patient's survival and quality of life.

## Results

### Postoperative outcomes

In 70 operated patients, the incision was all stage I healed after surgery, and the tumors all achieved macroscopic complete resection. Two patients with adenoid cystic carcinoma had positive intraoperative frozen return margins, one patient had 7 intraoperatively implanted radioactive particles, another patient was treated with postoperative radiotherapy of 66 Gy, and the other patients had negative margins for each. Patients with nasoendoscopic surgery, a few of them have symptoms such as mild nasal blockage, nasal dryness, and epistaxis after surgery, which can be relieved by nasal irrigation and symptomatic care. Among patients undergoing open surgery, 1 patient experienced slight diplopia except for a neck scar, which had no obvious impact on daily life and was not further treated. The patient who underwent a combined nasoendoscopic NPC resection via a parapharyngeal space approach had various degrees of incomplete paralysis of the mandibular ramus of the facial nerve, which normalized around 3 months postoperatively. None of the patients had dysphagia or dyspnea. The Standard Swallowing Function Assessment scale (SSA) was used to evaluate the patients' postoperative swallowing function, and the average score was 20 points, and the patients' postoperative swallowing function was good.

### Survival outcomes

**Figure 8:**
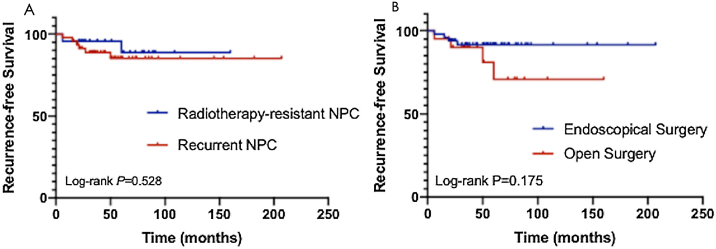
Subgroup survival analysis of patients with different pathological types and different surgical procedures. (A) The overall survival rate of NPC cases with recurrence after radiotherapy and those not sensitive to radiotherapy; (B) The overall survival rate of NPC cases who had nasoendoscopic surgery and open surgery.

Complete follow-up was available for 53 of 70 patients, including 10 with local recurrence and 4 with distant metastasis. The 3-year survival rate was 94.0%, and the 5-year overall survival rate was 88.7% (Fig. 8A). The 3-year recurrence free survival rate was 91.1%, and the 5-year recurrence free survival rate was 85.9% (Fig. 8B). After Kaplan Meier survival analysis, TNM stage and age at onset (grouped by <55 years) had a significant prognostic impact with *p* < 0.05.

A subgroup analysis of NPC cases with recurrence after radiotherapy and those not sensitive to radiotherapy found that the overall survival rate was 84.2% at 3 years and 78.6% at 5 years after surgical treatment for recurrent NPC cases. The overall survival rate was 83.3% at 3 years and 71.4% at 5 years after surgery for NPC of the radiotherapy insensitive type (Fig. 8A). Patients who had nasoendoscopic surgery had a higher survival rate than those who had open surgery, but the results were not statistically significant (Fig. 8B). COX multivariate regression analysis was performed for all risk factors of the patients, and the items with *p* < 0.05 were age, T stage. It can be concluded that tumor T stage and age higher than 55 years are risk factors for patient outcome, while gender, pathological type, N stage, preoperative chemoradiotherapy, postoperative chemoradiotherapy, family history, smoking history, drinking history, and surgical approach are not significantly associated with prognosis.

## Discussion

Radiotherapy combined with chemotherapy is preferred for the initial diagnosis and treatment of nasopharyngeal carcinoma. However, there are still 7%–15% cases in which the tumor remains or recurs after radiotherapy. Tumor recurrence occurs in approximately 10%–40% of patients within 1–2 years after initial radiotherapy.^9^, ^10^ There is no accepted standard of care for the management of recurrent nasopharyngeal carcinoma. For the management of recurrent nasopharyngeal carcinoma, there are guideline recommendations: a Multidisciplinary Treatment (MDT) model. Individualized treatment regimens are rationally formulated based on the patient's general condition, the size, location, stage, and interval between recurrences.^11^ Locoregional recurrence nasopharyngeal carcinoma that can meet the indications for surgery is the treatment of choice, and postoperative radiotherapy should be supplemented in those with positive surgical margins. In those who are unable to tolerate surgery or who are unresectable by surgery, re course radiotherapy should be administered. Chemotherapy, targeted therapy, and immunotherapy may be considered for those who refuse surgery and radiotherapy. For locoregional recurrence combined with metastasis nasopharyngeal carcinoma, radiotherapy of the recurrent lesion was considered only after the metastases achieved good control. Patients without indications for surgery and radiotherapy were considered for enrollment in the clinical trial.

Surgical treatment as an important treatment for recurrence after radiotherapy for nasopharyngeal carcinoma, previous open approaches have been used as the main surgical procedures such as: external rotation approach to the maxilla, infratemporal fossa approach, transmaxillary sinus approach, transhard palate approach, midfacial valgus approach with nasal incision, and mandibulotomy.^12^, ^13^ However, open access surgery is highly traumatic, and at the same time, it will cause postoperative facial scarring and other complications in patients, affecting the quality of life of patients. In recent years, with the continuous research on the anatomy of the endoscopic skull base, as well as the increasingly refined endoscopic equipment and instruments, and increasingly refined surgical techniques, the transnasal endoscopic technique has become a popular surgical modality for the surgical treatment of recurrent nasopharyngeal carcinoma. Scholars have reported high-quality evidence from clinical studies that surgery is superior to re course radiotherapy for patients who can undergo surgical resection after the evaluation of locoregional recurrence nasopharyngeal carcinoma.^7^ However, different groups have reported on the use of nasoendoscopic surgery for early-stage resectable nasopharyngeal carcinoma and salvage surgery for recurrent nasopharyngeal carcinoma, and the results of these studies have all shown the good therapeutic value of nasoendoscopic surgery for nasopharyngeal carcinoma.^8^, ^14^, ^15^, ^16^

However, the identification and protection of internal carotid artery in nasopharyngeal carcinoma surgery under nasal endoscopy is a technical difficulty. On the one hand, it requires complete nasal endoscopic instruments, navigation equipment, interventional embolization preparation of internal carotid artery during operation, hybrid operating room and other high hardware conditions. On the other hand, it requires high surgical technology and experience of the operator and his team. Therefore, only large medical centers can complete the surgical treatment of nasopharyngeal carcinoma, which puts forward a high threshold for the wide development of surgical treatment. This study developed and designed a new approach through the parapharyngeal space combined with nasal endoscopy. Seven patients with recurrent nasopharyngeal carcinoma and radiotherapy insensitive nasopharyngeal carcinoma were treated with new operation and achieved good prognosis. This method has little dependence on instruments and equipment, and it is relatively difficult to dissect the internal carotid artery up to the parapharyngeal segment through the neck. Surgical manipulation is performed under direct vision, and it is easier to locate the internal carotid artery with the aid of ultrasound Doppler. The submental flap protects the exposed internal carotid artery and the nasopharynx wound after dissection and reduces the risk of patient death from massive hemorrhage from internal carotid artery rupture. Comparing the surgical modalities of temporalis flap repair, the submental flap had less local inflammatory exudation, less scabbing, and quick postoperative recovery. Surgical procedure dissection of the parapharyngeal space and retropharyngeal lymph nodes was performed while removing the nasopharyngeal lesion. The nasal endoscopic field of view has the advantages of good mirror lighting conditions, close observation, magnification effect, and shows clear anatomical structures. The nasopharynx lesion was completely resected combining the advantages of the view of the nasoendoscope. Seven patients, all uneventfully revealed the glossopharyngeal, accessory, vagal, hypoglossal nerves in the parapharyngeal space, and given protection, the fat inside the carotid sheath and the lymph node tissue were dissected. After resection of the tumor, using the access from the parapharyngeal space, it was easy to divert the submental perforator flap superiorly to the nasopharynx and make a revision of the surgical wound. Seven patients had stage I healing of the incision and nasopharynx. All had a slight paresis of the mandibular ramus of the facial nerve postoperatively and recovered completely around 3 months postoperatively.

More than 95% of cases in the pathologic type of the nasopharynx are nonkeratinizing carcinomas, whereas adenocarcinoma is an uncommon type in the case type of nasopharyngeal carcinoma. The literature reports only about 2% of adenocarcinomas of minor salivary gland origin arising in the nasopharynx.^17^ Of 31,791 nasopharyngeal carcinomas statistically reported in the literature, only 153 (0.48%) were adenocarcinomas.^18^ For this reason, fewer studies have been reported for salivary gland nasopharyngeal carcinoma. Treatment options for nasopharyngeal adenocarcinoma there is currently no uniform standard in the academic community, and most recommendations are based on surgical treatment with adjuvant intraoperative radiotherapy. The MDT pattern was taken for this subset of cases in the enrollment pathology of our study. The surgical plan was decided by discussion of preoperative pathological diagnosis, imaging evaluation, and so on, after which the postoperative radiotherapy was decided based on the resection of the lesion during surgery. With the comprehensive treatment regimen of surgery and postoperative radiotherapy in our study, the 2-year overall survival rate of 93.7% and the 5-year overall survival rate of 71.4% after surgery for the radiotherapy insensitive type of nasopharyngeal carcinoma were higher than the 5-year survival rate of adenocarcinoma reported in the literature (58.4%). This may be related to the relatively early stage of our included patients and the small sample size.

## Conclusions

The comprehensive treatment of recurrent nasopharyngeal carcinoma and radiotherapy insensitive nasopharyngeal carcinoma with surgery can lead to a relatively good prognosis of survival. This study adopted the MDT approach to develop an individualized surgical plan for different patients, and the surgical approach of an external cervical approach combined with nasoendoscopy provided a new surgical reference for the surgical treatment of nasopharyngeal carcinoma. Because of the limited sample size of the cases reviewed in this study, further exploration of surgical treatment effects will be required in the future to expand the sample size, refine the staging of NPC, and type of cases.

## Ethics statement

The study protocol was approved by the Institutional Review Board of Beijing Tongren Hospital of Capital Medical University, and patient approval or informed consent was required for the review of the patients’ medical records. All procedures performed in studies involving human participants were in accordance with the ethical standards of the institutional and/or national research committee and with the World Medical Association Declaration of Helsinki (version 2008) and the additional requirements. Informed consent was obtained from all individual participants included in the study.

## Funding

This work was supported by the Key R&D Program of China (nº 2020YFB1312805), Capital Health Research and Development of Special Fund (nº 2022-1-2051), Priming Scientific Research Foundation for the Junior Researcher in Beijing Tongren Hospital, Capital Medical University (2021-YJJ-ZZL-018), National Natural Science Foundation of China (nº 82101187, 82002880), Beijing Municipal Administration of Hospitals Incubating Program (PX2021008), Beijing Hospitals Authority Youth Programme (QML20200205).

## Conflicts of interest

The authors declare that the research was conducted in the absence of any commercial or financial relationships that could be construed as a potential conflict of interest.
